# Amino acids for the prevention of acute kidney injury after cardiac surgery: a narrative review of trials and meta-analyses

**DOI:** 10.1186/s13019-026-04540-9

**Published:** 2026-07-21

**Authors:** Rolf Svedjeholm, Jonas Holm, Farkas Vanky

**Affiliations:** 1 Department of Health, Medicine and Caring Sciences, Unit of Cardiovascular Medicine, Linköping University, Linköping, Sweden; 2 Clinical Department of Thoracic and Vascular Surgery, Linköping University Hospital, Linköping, Sweden

**Keywords:** Acute kidney injury, Cardiac surgery, Amino acids, Glutamate, Glutamine, Renal functional reserve, Myocardial metabolism, Postoperative complications

## Abstract

Acute kidney injury (AKI) is one of the most common serious complications after cardiac surgery. Although many causes and risk factors have been identified, effective measures to prevent AKI after cardiac surgery are still lacking. Recent randomized trials and meta-analyses suggest a potential role for amino acids in preventing AKI after cardiac surgery. The growing interest in this topic is reflected in the number of recently published meta-analyses, which approaches that of adequately designed clinical trials addressing this subject. However, close examination of these meta-analyses reveals substantial heterogeneity among the included trials, particularly with respect to the selection of amino acids, dosages used, and proposed mechanisms of action. In this review, we describe the putative mechanisms of action, detail the major sources of heterogeneity and identify key gaps that need to be addressed to clarify the role of amino acids in preventing AKI following cardiac surgery.

## Background

Cardiac surgery is associated with a low mortality rate and favorable long-term outcomes; however, transient renal failure is not uncommon and was initially considered a minor complication. One reason for this perception is that, for a long time, it was unclear how acute kidney injury (AKI) after cardiac surgery should be defined. The introduction of standardized criteria (RIFLE, AKIN, KDIGO) enabled studies showing that AKI, already at stage I, is associated with increased postoperative morbidity and mortality (Table 1) [1–7]. AKI is now recognized as one of the most common serious complications after cardiac surgery, occurring in 7–40% of patients depending on the definition used and patient selection [4, 8]. In the United States, the additional health care costs attributable to AKI after cardiac surgery are estimated at approximately one billion dollars annually [8].

**Table 1:** RIFLE, AKIN and KDIGO criteria for AKI

Stage	RIFLE	AKIN	KDIGO	Urine output (same for all)
**Risk/stage 1**	≥50% increase of s-Cr ≤ 7 days or ≥25% decrease of GFR	≥26.5 µmol/L (0.3 mg/dL) increase of s-Cr ≤ 48 h or ≥50% increase of s-Cr ≤ 7 days	≥26.5 µmol/L (0.3 mg/dL) increase of s-Cr ≤48 h or ≥50% increase of s-Cr ≤ 7 days	< 0.5 mL/kg/h ≥ 6 h
**Injury/stage 2**	≥100% increase of s-Cr or ≥50% decrease of GFR	≥100% increase of s-Cr	≥100% increase of s-Cr	< 0.5 mL/kg/h ≥12 h
**Failure/stage 3**	≥200% increase of s-Cr increase of s-Cr to ≥ 354 µmol/L (4 mg/dL) with an acute increase of 44 µmol/L (0.5 mg/dL) or ≥75% decrease of GFR	≥200% increase of s-Cr or increase of s-Cr to ≥ 354 µmol/L (4 mg/dL) with an acute increase of 44 µmol/L (0.5 mg/dL) or Initiation of KRT	≥200% increase of s-Cr or increase of s-Cr to ≥ 354 µmol/L (4 mg/dL) or Initiation of KRT	< 0.3 mL/kg/h ≥24 h or Anuria ≥ 12 h
**Loss**	Loss of kidney function > 4 weeks			
**End-stage**	Loss of kidney function > 3 months			

Change in s-Cr or GFR from baseline for staging of AKI. GFR = Glomerular Filtration Rate, KRT = Kidney Replacement Therapy, s-Cr = serum Creatinine

Several causes and risk factors for AKI after cardiac surgery have been identified: however, aside from general supportive measures – such as optimizing fluid status, maintaining perfusion pressure, and avoiding nephrotoxic substances – there are currently no evidence-based measures to prevent AKI [2, 3, 9]. Since adequate kidney perfusion is crucial, inotropic agents have been widely used to enhance circulation [10, 11]. However, there is no evidence that these drugs prevent AKI; on the contrary, some studies suggest that they may be harmful [11, 12]. A possible explanation is that inotropic drugs increase the oxygen demand in both the heart and peripheral tissues [13].

Amino acids have recently emerged as candidates for the prevention of AKI following reports that infusion of amino acids was associated with a significantly lower incidence of AKI after cardiac surgery [10, 14]. In addition, we identified four meta-analyses on this topic published from October 2024 to July 2025 [15–18]. Notably, one of our own publications was included in two of the meta-analyses [19]. Further review revealed substantial heterogeneity among the included trials, particularly regarding amino acid selection, dosing, and proposed mechanisms of action. Here, we provide an overview of these discrepancies, with focus on the two largest studies.

## Methods

We scrutinized the trials on amino acid infusions in cardiac surgical patients included in four meta-analyses published from October 2024 to July 2025 (Tables 2, 3, 4 and 5) [15–18].

**Table 2:** Trials included in meta-analyses published from October 2024 - July 2025

Meta-analysis	Landoni et al. [10]	Holm et al. [19]	Weiss et al. [61]	Pu et al. [8]	Kazawa et al. [14]
Pruna et al. [18]	Yes	-	-	Yes	Yes
Jiang et al. [15]	Yes	Yes	Yes	Yes	Yes
Majeed et al. [16]	Yes	Yes	Yes	Yes	Yes
Khan et al. [17]	Yes	-	-	Yes	Yes

**Table 3:** Study design of trials included in the meta-analyses from October 2024 - July 2025

	Landoni et al. [10]	Holm et al. [19]	Weiss et al. [61]	Pu et al. [8]	Kazawa et al. [14]
Design	Prospective RCT	Retrospective analysis of pooled data from 2 RCT	Prospective RCT pilot	Prospective RCT pilot	Prospective RCT pilot
Blinding	Double blind	Double blind	Double blind	No	Assessor blinded
Multicenter	Yes	Yes	No	No	No
Number of patients studied	3511 (1759 vs 1752)	791 (391 vs 400)	63 (31 vs 32)	68 (33 vs 35)	65 (33 vs 32)
Patients	Elective mixed cardiac surgery with CPB	CABG ± valve with CPB	Mixed cardiac surgery with CPB	Mixed cardiac surgery with CPB	Elective Aortic surgery with CPB
Specific inclusion criteria	No	Without diabetes Glutamics I: ACS Glutamics II: LVEF ≤0.30 or EuroSCORE II ≥3.0 with cardiac risk factor	Urinary [TIMP2]*[GFBP7] ≥0.3 4 h after surgery	Expected CPB>60 min + GFR 90-20 mL/min	No
Chronic kidney disease excluded if:	GFR<30 mL/min or KRT	GFR<30 mL/min or KRT	GFR< 30 mL/min or KRT	GFR <20 mL/min or KRT	GFR<15 mL/min or KRT
Stratification	Site	Isolated CABG / CABG + valve		GFR above or below 30 mL/min	Age, GFR, thoracoabdominal aortic surgery
Primary end-point	AKI KDIGO s-Cr­↑ ≥26.5 µmol/L≤48 h or s-Cr­ ↑ ≥50% ≤7 days	AKI RIFLE s-Cr↑ ≥50% during hospital stay	Urinary [TIMP2]*IGFBP7] after 12h infusion	Duration of renal dysfunction ≥s-Cr 168 µmol/L or AKI during hospital stay	AKI KDIGO s-Cr­­↑ ≥26.5 mmol/L ≤48 h or s-Cr­­↑ ≥50% ≤7 days or Urine output criteria
AKI criteria	KDIGO: s-Cr­↑ 26.5 µmol/L≤48 h or s-Cr­↑ 50% ≤7 days without urine output criteria	RIFLE: s-Cr↑ ≥50% during hospital stay without urine output criteria	KDIGO: ≤72 h-Cr↑­ 26.5 mmol/L≤48 h or s-Cr↑­ 50% ≤72 h without urine output criteria	AKI duration: KDIGO AKI incidence: s-Cr↑ ­ ≥50% without urine output criteria	KDIGO: s-Cr↑ ≥ 26.5­µmol/L ≤48 h or s-Cr­↑ ≥ 50% ≤7 days+ urine output criteria

ACS = Acute Coronary Syndrome, AKI = Acute Kidney Injury, CABG = Coronary Artery Bypass Grafting, CPB = Cardiopulmonary Bypass, GFR = Glomerular Filtration Rate, KRT = Kidney Replacement Therapy, LVEF = Left Ventricular Ejection Fraction, RCT = Randomized Controlled Trial,  s-Cr = serum Creatinine

Two trials cited by Jiang et al. were excluded from our review, as one was conducted in critically ill ICU patients and the other evaluated kidney perfusion with a Histidine-Tryptophan-Ketoglutarate solution during thoracoabdominal aortic surgery [15, 20, 21]. Three trials assessed by Pruna et al. for AKI were excluded from this review because they were conducted in urologic, orthopedic, and obstetric surgery [22–24]. Notably, the authors of the meta-analysis by Pruna et al. were also involved in the trial by Landoni et al., while as previously disclosed, the authors of this overview, were involved in the trial by Holm et al. [10, 18, 19].

## Mechanisms

The reviewed publications propose different mechanisms to explain the preventive effect. Three of them are based on the concept that protein or amino acid loading stimulates the recruitment of renal functional reserve (RFR), thereby protecting the kidneys [10, 25]. The other mechanisms involving single amino acids, glutamate and glutamine, are described below.

### Amino acid loading for recruitment of renal functional reserve (Pu et al. [8], Kazawa et al. [14], Landoni et al. [10])

It has long been recognized that the kidneys possess a significant reserve capacity, which enables organ donation in kidney transplantation. In 1983, Bosch et al. demonstrated that protein loading increases glomerular filtration rate (GFR) in humans and introduced the concept of renal functional reserve (RFR), defined as the kidneys’ ability to increase GFR in response to an amino acid load [25]. In a sheep model, recruitment of RFR with intravenous amino acid infusion was associated with improved cortical and medullary oxygenation [26]. Increased nephron plasma flow and oxygenation are mediated by vasodilation at the preglomerular arteriolar level and modulation of tubuloglomerular feedback. However, the mechanisms underlying RFR recruitment are complex and not fully understood [27]. While glucagon appears to play a central role, it alone cannot explain the effect on the kidneys, which seems to depend on an interplay between endocrine (Glucagon, Vasopressin, Growth hormone/Insulin-like growth factor 1), paracrine (Nitric oxide and vasodilating prostaglandins), and metabolic factors [27].

Resting GFR and plasma creatinine levels typically remain normal until approximately half of the nephrons are lost [28]. The response to intravenous amino acids can be used to detect patients at risk of developing kidney disease [27]. RFR declines with kidney disease, pregnancy, and advancing age, and reduced reserve capacity is associated with an increased risk of AKI [29].

Early speculation suggested that recruitment of RFR might be used protect the kidneys. Large observational studies reported an association between high protein intake and reduced mortality in critically ill patients [30–32]. In a randomized controlled trial (RCT) involving 474 adult patients expected to stay in intensive care ≥ 2 days, amino acid loading 2 g/kg/day up to 100 g/day improved GFR and urine output compared to standard care, but did not significantly affect the primary endpoint – the duration of renal dysfunction defined as s-Cr ≥168 µmol/L. Additionally, serum urea levels increased in the amino acid group [20]. A subsequent post hoc analysis found that intravenous amino acid loading was associated with higher GFR and lower 90-day mortality in patients with normal kidney function at study inclusion [33].

In cardiac surgery, Jeppsson et al. found that amino acid infusion shortly after coronary artery bypass grafting (CABG) elevated GFR, improved renal blood flow, and enhanced kidney oxygenation [34]. A non-blinded single-center cohort study of 110 adult patients undergoing mixed procedures found that a high oral protein intake the day before surgery was associated with better GFR but did not reduce the incidence of AKI [28]. Administration of oral protein the day before surgery may have missed the optimal time window to confer renal protection. Three subsequent RCTs investigating RFR recruitment to prevent AKI, using intravenous amino acid infusions intraoperatively and postoperatively are described below (Table 2) [8, 10, 14].

Pu et al. studied 69 adult patients undergoing mixed cardiac surgery with an expected pump run exceeding 1 h and a preoperative GFR between 20 and 90 mL mL/min/1.73 m^2^ in a small, single-center, unblinded pilot RCT [8]. Patients were randomized to receive either amino acid infusion or standard care. Despite randomization, baseline characteristics were somewhat imbalanced with no apparent advantage for the amino acid group. By chance a significantly higher proportion of females was allocated to this group. The infusion of mixed amino acids was initiated after induction of anesthesia and continued until discharge from the intensive care unit (ICU). The dose 2 g/kg/bw up to 100 g/day was adjusted if other sources of protein were provided. The primary endpoint was clinically significant renal dysfunction, defined as s-Cr ≥168 µmol/L. Neither the primary endpoint nor the incidence of AKI differed significantly between groups. In addition, a significant increase in blood urea was observed. However, the duration of AKI, defined by KDIGO, was shorter in the amino acid group. Moreover, these patients had better GFR and urine output. (Tables 3, 4 and 5).

**Table 4:** Interventions used in trials included in the meta-analyses from October 2024 - July 2025

	Landoni et al. [10]	Holm et al. [19]	Weiss et al. [61]	Pu et al. [8]	Kazawa et al. [14]
Intervention	Isopuramin 10%^®^(1000 mL)	Glutamic acid 0.125M (500 mL)	Dipeptiven^®^	Synthamine 17 electrolyte free^®^ (1000 mL)	Amiparen^®^ (200 mL)
Amino acids	Alanine 7.0g; Arginine 11.7g; Cysteine Chlorhydrate 0.20g; Glycine 6.4g; Histidine 5.1g; Isoleucine 8.0g. Leucine 10.6g; Lysine Acetate 16.3g; Methionine 7.2g; Phenylalanine 7.9g; Threonine 9.5g; Tryptophan 3.2g; Tyrosine 0.30g; Valine 11.6g	Glutamic acid 9.2 g	L-Alanyl-Glutamine 20% concentration 100 mL = 13.4 g L-glutamine and 8.2 g L-alaninedissolved in equal amounts of saline	Alanine 20.7g; Arginine 11.5 g; Glycine 10.3g; Histidine 4.8g;Isoleucine 6g; Leucine 7.3g; Lysine 5.8g;Methionine 4.0g;Phenylalanine 5.6g:Proline 6.8g;Serine 5g; Threonine 4.2g; Tryptophan1.8g; Tyrosine 0.4g; Valine 5.8g	Alanine1.6g; Arginine 2.1g; Aspartate 0.2g; Cysteine 0.2g; Glutamate 0.2g; Glycine 1.18g; Histidine 1g; Isoleucine 1.6g; Leucine 2.8g: Lysine-acetat 2.96h; Lysine 2.1g: Methionine 0.78g; Phenylalanine 1.4g; Proline 1g; Serine 0.6g; Threonine 1.14g; Tryptophan 0.4g; Tyrosine 0.1g;Valine 1.6g;
Control/Placebo	Ringer	Saline	Saline	Standard care	Standard care, amino acids not given to controls for 3 days
Start of Amino acids	Admission to operation room	Intraoperatively before release of X-Clamp	Postop 4 h after CPB	After induction of anesthesia	After induction of anesthesia
Duration	up to 3 days or until discharge from ICU, KRT or death	2.5 - 3 h	12 h	until discharge from ICU	up to 3 days
Dose	2g/kg/BW day up to max 100g/day	1.65 ml/kg/h of 0.125M glutamic acid solution for 2.5-3 h	0.5g/kg/BW/ during 12 h	100 g/day	60 g/day
Dose of amino acids adjusted for other sources of amino acids	Yes, reduced to 2g/kg/day if exceeding 2.5g/kg/day	No	No	Yes, reduced to 2g/kg/day if exceeding 2.5g/kg/day	No
Amino acid dose 70 kg patient first day	100 g	6 g	35 g	100 g	60 g
Glutamic acid dose 70 kg patient	0	6 g	0	0	0.6 g
Glutamine dose 70 kg patient	0	0	21.7 g	0	0
Adverse effects	s-Urea increased?	No difference between groups	No difference between groups	s-Urea increased	s-Urea increased
Urea active vs control	53 (41-71) vs 41 (31-54) mg/mL (p=?) Median (IQR)	?	?	11.4 ± 9.0 vs 8.4 ± 8.3 mmol/L (p=0.028)	12.8 (95%CI 11.7 - 14.0) vs 10.8 (95%CI 9.3-12.3) mmol/L (p=0.02)

BW = body weight, CPB = Cardiopulmonary Bypass, ICU = Intensive Care Unit, KRT = Kidney Replacement Therapy, X-clamp = aortic cross-clamp

Kazawa et al. studied 65 patients undergoing elective thoracic or thoracoabdominal aortic surgery on cardiopulmonary bypass (CPB) in a single-center, patient-assessor-blinded pilot RCT. Stratification was done based on the extent of aortic surgery, age, and GFR. Patients were randomized to receive either amino acid infusion or standard care. The infusion was initiated after induction of anesthesia at a dose of 60 g/day for up to 3 days. The primary endpoint was AKI defined by KDIGO criteria, including urine output. The incidence of AKI was significantly lower in the intervention group (30.3% vs. 56.2%), with an adjusted hazard ratio of 0.44 (0.20–0.95; *p* = 0.04). This finding was primarily driven by differences in urine output, as AKI incidence based on creatinine criteria did not differ significantly between groups (Tables 3, 4 and 5).

Landoni et al. included 3,511 patients in the PROTECTION trial, a prospective, randomized, double-blinded study conducted at 22 sites across three countries, 19 of which were located in Italy [10]. Patients underwent elective cardiac surgery, fairly evenly distributed between CABG, aortic, and mitral valve surgery. A total of 1,759 patients were randomized to receive amino acid infusion and 1,752 to receive Ringer’s solution at the same infusion rate. The dose of amino acids was 2 g/kg /BW per day up to a maximum of 100 g/day. The infusion was initiated after admission to the operating room and continued up to three days or until the patient left the ICU, started kidney replacement therapy (KRT), or died. The dose was reduced to account for other sources of protein, targeting 2 g/kg per day if the total intake exceeded 2.5 g/kg per day. The median infusion durations in the intervention and control groups were 30 h and 31 h, respectively, resulting in median amino acid doses of 126 g and 127 g, respectively. The primary endpoint, AKI according to KDIGO, based on creatinine values alone, was significantly lower in the treatment group (26.9% vs. 31.7%; Relative Risk (RR) 0.85 [95% CI 0.77–0.94; *p* = 0.002]). The significant effect was demonstrated in elective patients in NYHA class I–II, who comprised 79% of the study population (RR 0.82, 95% CI 0.72–0.93; *p* = 0.002). No significant effect was observed among patients in NYHA class III–IV (RR 0.93, 95% CI 0.78–1.10; *p* = 0.41). No significant differences in KRT, survival, or quality of life were observed at 30, 90, or 180 days of follow-up (Tables 3, 4 and 5).

In their editorial, Ostermann and Shaw noted that 336 patients underwent hemofiltration during CPB, which may complicate interpretation of postoperative creatinine levels. However, as hemofiltration was equally distributed between the amino acid group and the control group it is unlikely to have biased the results in favor of either group [35]. Although the study is unique and well-designed, it remains uncertain to what extent the results reflect a functional effect or true protection against tubular kidney injury. The incidence of AKI stage 3 suggests a genuine effect; however, analysis of tubular kidney injury markers would have been desirable. In addition, the editorial called for data on incident or progressive chronic kidney disease beyond 90 days, as well as a health economic analysis [36]. A subsequent cost-effectiveness evaluation found that perioperative amino acid therapy in cardiac surgery was associated with decreased healthcare costs [37].

Some may argue that routine clinical nutrition with amino acids is sufficient. However, a real-world study from Japan involving 30,751 surgical patients – including 4,434 undergoing cardiovascular surgery – found no difference in postoperative AKI incidence between patients who received amino acids on the day of cardiovascular or non-cardiovascular surgery and those who did not. These results should be interpreted with caution, as this was not a randomized controlled trial and, notably, few patients received amino acid doses comparable to those used in the PROTECTION trial [38].

### Enhanced recovery of oxidative myocardial metabolism with glutamate (Holm et al. [19])

The kidneys depend on adequate blood flow, and since myocardial dysfunction after cardiac surgery is one of the leading causes of AKI, glutamate infusion could indirectly prevent AKI by enhancing post-ischemic recovery of cardiac metabolism and function [39, 40].

Cardiac metabolism and function are closely interconnected. During myocardial ischemia, cytosolic glutamate and aspartate levels decline, mitochondrial citric acid cycle intermediates are depleted, and ATP levels fall, resulting in contractile dysfunction [39, 40]. During reperfusion, when myocardial blood flow is restored, myocardial oxygen utilization is compromised due to depletion of mitochondrial citric acid cycle intermediates, and cardiac function remains transiently impaired [40, 41]. In severe cases, ischemia-reperfusion injury results in cell death and myocardial infarction.

While not significant as energy sources, amino acids linked to the Malate-Aspartate shuttle, such as glutamate and aspartate, play a key role in cardiac metabolism in association with ischemia [42]. During ischemia, they facilitate anaerobic metabolism via the malate-aspartate shuttle. After ischemia, they promote recovery of oxidative metabolism by restoring mitochondrial citric acid cycle metabolites lost during ischemia. In animal studies, the administration of glutamate or aspartate promotes normalization of oxidative metabolism and recovery of contractile function [43]. The effect is attenuated in the diabetic heart due to downregulation of the mitochondrial glutamate transporter EAAT1, which is essential for the function of the malate-aspartate shuttle [44, 45].

The human heart adapts to ischemia by increasing glutamate uptake [46, 47]. High fractional extraction of glutamate has been observed shortly after CABG, preceding normalization of myocardial metabolism. In fact, only oxygen displayed a higher fractional rate than glutamate. Furthermore, a strong correlation was observed between arterial glutamate levels and myocardial uptake, indicating a relative substrate deficiency [48]. Intravenous infusion of glutamate at this stage increased plasma levels and cardiac uptake of glutamate, accompanied by a normalization of myocardial metabolism and improved cardiac function [49, 50]. Consistent with animal studies, synthesis of citric acid cycle intermediates is the primary fate of ^13^C-labeled glutamate extracted by the human heart after CABG [51].

Despite advances in myocardial protection during cardiac surgery, virtually all hearts exhibit some degree of dysfunction during reperfusion. The impairment of contractility is usually mild and rapidly transient but may be severe enough to require pharmacological or mechanical circulatory support, particularly in patients who sustain intraoperative myocardial infarction or have limited preoperative cardiac reserve.

Based on encouraging clinical experience with intravenous glutamate infusion in critically ill and high-risk patients, we initiated clinical trials [52–54]. GLUTAMICS I was a prospective randomized, double-blind trial in 861 patients, investigating whether glutamate infusion prevents myocardial infarction, postoperative heart failure, and mortality after CABG for acute coronary syndrome [55]. The protective effect could not be confirmed, partly because shifts in cardiological practice led to a high proportion of low-risk patients being referred for urgent CABG. These patients had favorable outcomes irrespective of intervention, and some degree of cardiac metabolic disturbance is required for glutamate infusion to make a difference [49, 50, 56]. Notably, a reduced incidence of postoperative heart failure leading to death or prolonged ICU stay was observed in the cohort originally targeted for the trial – patients with unstable angina in CCS class IV [54]. These findings supported further evaluation of glutamate infusion in patients at risk of postoperative heart failure.

To avoid inclusion of low-risk patients, GLUTAMICS II enrolled 303 patients with at least a moderately increased risk of developing postoperative heart failure based on preoperative cardiac function or EuroSCORE II. The primary endpoint was the postoperative increase in plasma N-terminal pro-brain natriuretic peptide (NT-proBNP), a biomarker of heart failure that also rises in renal failure due to reduced clearance [57]. However, the proportion of patients with diabetes – reported to have a blunted glutamate response – unexpectedly increased to 47%, nearly double that in the cohort used for the sample size calculation. In patients without diabetes, the postoperative increase in NT-proBNP was significantly lower in the glutamate group (4503 ± 4846 ng/L vs. 6824 ± 5671 ng/L; *p* = 0.007). In addition, the incidence of AKI (RIFLE stage Risk or higher) was significantly lower with glutamate (11% vs. 29%; *p* = 0.005).

A pooled analysis of all 791 patients without diabetes enrolled in GLUTAMICS I and II showed a lower incidence of AKI in the glutamate group, both at RIFLE stage I or higher (4.9% vs. 10.0%; *p* = 0.007) and RIFLE stage II or higher (1.0% vs. 4.0%; *p* = 0.01) [19]. Although the total amino acid dose was insufficient to induce RFR recruitment, the study was included in two meta-analyses (Table 2) [15, 16].

Limited commercial interest and concerns about glutamate-related excitotoxicity in the brain are the main reasons for the small number of human studies. An infusion rate of 1.65 mL/kg BW/h of a 0.125 M glutamic acid solution, resulting in a two- to three-fold increase in arterial whole-blood glutamate levels, was identified as the minimum dose sufficient to optimize, or saturate, myocardial glutamate uptake shortly after CABG [58]. In clinical practice, intravenous glutamate infusion was first used in isolated cases of postoperative heart failure refractory to conventional therapy [52]. Based on encouraging experience with these critically ill patients, its use was extended to prophylactic treatment in high-risk patients [54]. Monitoring of our clinical experience together with two clinical trials – including a substudy on a biomarker of subclinical neurological injury (S100B) – has not revealed any evidence of neurological or other adverse events [55, 57, 59, 60].

## Protective effect of glutamine (Weiss et al. [61])

Glutamine is the most abundant free amino acid in humans and is considered a conditionally essential during critical illness [62]. Glutamine has therefore attracted considerable interest in clinical nutrition, with studies suggesting potential benefits for the heart, kidneys, intestines, and the immune system [62]. Glutamine is reported to enhance mitochondrial function and reduce apoptosis in tubular epithelial cells of mouse kidneys [63]. Glutamine is vital for maintaining the integrity of the intestinal barrier [64]. Glutamine has also been reported to protect the heart against ischemia [65]. Anti-inflammatory, antioxidant, and anti-apoptotic effects are considered to be mediated by the expression of glutathione, heme oxygenase-1, and heat shock proteins [66].

Due to stability issues, glutamine is typically infused as an alanine-glutamine dipeptide. Small, single-center RCTs have reported favorable effects of glutamine supplementation on clinical outcomes in critically ill patients. However, larger multicenter RCTs have reported increased mortality or trends toward increased mortality; accordingly, glutamine supplementation in ICU patients is not recommended by the American Society for Parenteral and Enteral Nutrition [67, 68].

Limited data are available on the effect of glutamine against AKI in cardiac surgery. Weiss et al. published a single-center pilot RCT in cardiac surgery patients deemed to be at increased risk of AKI [61]. The concentration of biomarker [TIMP2]*[IGFBP7] increases during tubular stress before tissue injury. Patients with a urinary [TIMP2]*[IGFBP7] ≥ 0.3 four hours after CPB were eligible for inclusion. Participants received either Saline or L-Alanyl-Glutamine (0.5 g/kg/BW) infused over 12 h. A total of 354 patients were screened, and 64 patients undergoing mixed cardiac surgery – including surgery for aortic dissections and endocarditis – were studied. The primary endpoint was [TIMP2]*[IGFBP7] after the 12-hour infusion period. The authors reported significant effects for the primary endpoint and for other markers of tubular kidney injury (KIM^1^ and NGAL), but not for the incidence of AKI at 72 h or for clinical outcomes during the 90-day follow-up. This trial was included in two meta-analyses [15, 16].

## Comparison of trials included in the meta-analyses

Two of the meta-analyses examined five trials evaluating amino acid infusions for prevention of AKI in cardiac surgery [8, 10, 14, 19, 61]. The other two meta-analyses included three of these trials (Table 2) [8, 10, 14]. Details of the individual trials are given in Tables 3, 4 and 5, and variables of particular interest related to study design, interventions, and study populations are commented below.

## Study design (Table 3)

Four studies (Landoni, Weiss, Pu, Kazawa) were prospective randomized controlled trials, whereas GLUTAMICS I-II by Holm was a retrospective analysis of pooled data from two randomized controlled trials. Three of the prospective trials (Weiss, Pu, Kazawa) were pilot studies (Table 3).

### Blinding

Three of the studies were double-blinded (Landoni, Holm, Weiss), one was assessor-blinded (Kazawa), and one was unblinded (Pu).

### Sites

Only two of the trials were multi-center studies (Landoni, Holm). The PROTECTION trial enrolled patients from 22 centers across three countries, whereas GLUTAMICS I-II recruited patients from five cardiac surgery centers in Sweden.

### Stratified randomization

Landoni stratified randomization by site, whereas Holm stratified patients by surgical procedure (isolated CABG vs. CABG with concomitant valve surgery). Pu stratified patients by baseline GFR above or below 30 mL/minute, and Kazawa stratified patients according to age, GFR, and extent of aortic surgery.

### Specific inclusion criteria

Holm enrolled patients with prespecified indicators suggesting an increased risk of postoperative heart failure (Table 3). Weiss included patients at an increased risk of AKI defined by urinary [TIMP2]*[IGFBP7] ≥ 0.3 four hours after surgery. Pu included patients with an expected CPB duration > 60 min who had a reduced GFR (20–90 mL/min). Kazawa included only patients undergoing elective thoracic or thoracoabdominal aortic replacement on CPB.

### Preoperative kidney function

Preoperative kidney disease is a risk factor for AKI [25], and all studies excluded patients with severe preoperative kidney dysfunction or those receiving KRT, as detailed in Table 3.

### Sample size

Three studies included fewer than 70 patients each (Weiss, Pu, Kazawa), whereas the other two, Landoni (*n* = 3511) and Holm (*n* = 791), accounted for almost 96% of the patients.

### Primary endpoint

AKI was the primary endpoint in three studies, but AKI criteria differed (Landoni, Holm, Kazawa). Two studies reported AKI as a secondary endpoint (Weiss, Pu). Weiss used urine [TIMP2]*[IGFBP7] measured after 12-hour infusion of amino acids or saline as the primary endpoint, whereas Pu used clinically significant renal dysfunction defined as s-Creatinine ≥ 168 µmol/L (Table 3).

### AKI criteria

Four studies applied KDIGO criteria (Landoni, Weiss, Pu, Kazawa), but only Kazawa used the full definition, including urine output. Using urine output criteria increases sensitivity and may allow earlier detection of AKI, but the clinical implications remain uncertain [69]. Notably, the significant difference reported by Kazawa was largely driven by the urine output data [14]. Landoni, Pu and Weiss used creatinine-based KDIGO criteria, with Weiss limiting assessment to the first 72 h after surgery. Holm reported AKI using creatinine-based RIFLE criteria.

### Interventions (Table 4)

**Table 5:** Patient data and outcomes in trials included in the meta-analyses from October 2024 - July 2025

	Landoni et al. [10]	Holm et al. [19]	Weiss et al. [61]	Pu et al. [8]	Kazawa et al. [14]
Age	66 vs 67 (median)	69 vs 70 (mean)	70 vs 67 (mean)	72 vs 71 (mean)	72 vs 71 (median)
Females %	30.1 vs 30.1	15.9 vs 18.3	6.1 vs 5.6	42.4 vs 13.9	51.5 vs 34.4
Diabetes %	17.6 vs 19.1	0 vs 0	12.9 vs 28.13	21.2 vs 13.9 (insulin)	12.1 vs 9.4
s-Creatinine μmol/L preoperatively	85 vs 83 (median)	97 vs 97 (mean)	88 vs 87 (mean)	114 vs 105 (mean)	70 vs 77 (median)
NYHA III-IV %	20.4 vs 20.4	75.2 vs 76.5	12.9 vs 6.26	17.5 vs 13.6	?
Urgent or Emergent patients %	0	90.8 vs 90.8	?	27.3 vs 41.7	0
Preoperative risk score	No	EuroSCORE 5.1 v 4.9* (mean)EuroSCORE II 3.6 v 3.7** (mean)	EuroSCORE 6 v 5 (median)	No	No
CABG %	34.8 vs 36.5	100 vs 100	45.2 vs 56.3	48.4 vs 75.0	0
Valve or other procedure %	83.2 vs 81.1	9.0 vs 9.8	54.8 vs 43.8	57.5 vs 36.1	0
Aortic surgery %	12.4 vs 14.1		6.45 vs 6.25		100 vs 100
Remark			2 Aortic dissections, 7 endocarditis		
X-clamp (min)	68 vs 69 (median)	56 vs 57 (mean)	78 vs 80 (median)	112 vs 136 (mean)	134 vs 167 (median)
CPB (min)	93 vs 94 (median)	88 vs 89 (mean)	117 vs 124 (median)	248 vs 286 (mean)	242 vs 287 (median)
Incidence AKI %	26.9 vs 31.7	4.9 vs 10.0	25.0 vs 35.5	9.0 vs 20.0	30.3 vs 56.2
Effect size RR	0.85 (95%CI 0.77-0.94) p=0.002	0.49 (95%CI 0.29-0.83) p=0.008	1.42 (95%CI 0.66-3.05) p=0.37	0.45 (95%CI 0.13-1.61) p=0.22	0.63 (95% CI 0.40-0.99) p=0.04***
KRT %	1.4 vs 1.9	0.5 vs 1.2	3.2 vs 0	12.1 vs 2.9	0 vs 9.4
Mortality ≤30 days %	2.8 vs 2.8	0.5 vs 1.0	12.9 vs 3.1	6.1 vs 2.8	0 vs 0

AKI = Acute Kidney Injury, CABG = Coronary Artery Bypass Grafting, CI = Confidence Interval, CPB = Cardiopulmonary Bypass, KRT = Kidney Replacement Therapy, NYHA = New York Heart Association class, RR = Relative Risk, s-Cr = serum Creatinine, X-clamp = aortic cross-clamp, * for 315 patients ** for 316 patients, *** reported as adjusted Hazard Ratios in the publication

### Selection of amino acids

Three studies targeting recruitment of RFR used mixed balanced amino acid solutions (Landoni, Pu, Kazawa). Two studies used single amino acids: glutamate (Holm) or the dipeptide L-Alanyl-Glutamine (Weiss). None of the other trials administered glutamate or glutamine, except for trace amounts of glutamate (and aspartate) in the trial by Kazawa.

The potential role of individual amino acids within mixed amino acid solutions remains to be clarified. For instance, branched-chain amino acids and arginine may activate signaling pathways involved in mitochondrial biogenesis, potentially conferring a survival benefit [70–72]. Arginine, ornithine, and aspartate may theoretically exert effects similar to glutamate through their roles in the malate–aspartate shuttle [39, 73]. Taurine may protect against tubular kidney injury due to its antioxidant and osmoregulatory properties [74]. The composition of amino acids solutions used are detailed in Table 4.

### Amino acid dose and duration of treatment

The duration of intervention ranged from 2.5 h to 3 days, with total amino acid doses for a 70 kg patient ranging from 6 g to 300 g. Two of the three studies citing recruitment of RFR as the purpose of the intervention typically infused 2 g/kg BW up to 100 g per day up to 3 days until discharge from the ICU (Landoni, Pu). The dose in these studies was modified to account for other sources of protein, as presented in Table 4. A secondary analysis of the PROTECTION trial did not demonstrate additional benefit from extending the infusion beyond 48 h; on the contrary, a potential adverse effect on survival could not be excluded. Therefore, infusions exceeding 48 h are not recommended [75]. The third study by Kazawa administered 60 g/day up to 3 days.

The amino acid dose and duration of treatment in trials using single amino acids were substantially lower. Approximately 6 g of glutamate was infused over 2.5–3 h, whereas a glutamine dose of 35 g was administered over 12 h [19, 61].

### Timing of the intervention

The optimal timing for administering amino acids in cardiac surgery remains to be established. Subgroup analysis suggested that the timing of intervention could influence the results, with intraoperative amino acid infusion more likely to succeed [15]. Four trials provided amino acids intraoperatively (Landoni, Holm, Pu, Kazawa), whereas one trial (Weiss) initiated the infusion postoperatively 4 h after termination of CPB.

### Control treatment

Three trials (Landoni, Holm, Weiss) used crystalloid solutions in control patients, whereas Pu and Kazawa used standard care.

### Postoperative urea levels

As expected, increased postoperative urea levels were reported in the trials using high amino acid doses sufficient to stimulate RFR (Table 4). Transient elevations in blood urea that resolve rapidly after discontinuation of amino acids infusions are likely innocuous. However, the impact of elevated blood urea in patients receiving amino acid infusions who subsequently develop AKI after cardiac surgery remains unclear. Pu et al. cautioned that future investigators should closely monitor blood urea levels [8].

### Adverse events

No adverse events related to the intervention was reported in any of the studies.

## Patients and outcomes (Table 5)

### Baseline characteristics

Overall, baseline characteristics were evenly balanced between the intervention and control groups. However, in the study by Pu, female sex was unevenly distributed (*p* < 0.05), with trends toward imbalance in urgent-emergent procedures, and the proportion of CABG procedures, but not in favor of the amino acid group.

### Procedures and preoperative risk scores

Underlying heart disease and the type of cardiac surgical procedure are known to influence the incidence of AKI and related biomarkers [76, 77]. CABG combined with valve procedures carries the highest reported risk of postoperative dialysis [76]. A homogeneous surgical population is therefore preferable. Urgent and emergent procedures are also associated with a higher risk for postoperative complications, including AKI [2]. As shown in Table 4, the type and acuity of procedures varied markedly across studies, and only two trials applied stratified randomization by surgical procedure (Holm, Kazawa). Preoperative risk scores such as EuroSCORE II predict postoperative mortality but are also associated with an increased risk of AKI [78, 79]. Only two studies (Holm, Weiss) reported preoperative risk scores.

### Incidence of AKI among controls

An unexpectedly high event rate in the control group may increase the risk of a Type I error. Across studies, the incidence of AKI in control groups ranged from 10% to 56.2%. In the study by Kazawa el al., the high incidence of AKI was at least partly explained by the surgical case mix, and the use of full KDIGO criteria to define AKI in that trial [14]. The lowest incidence was reported in the study by Holm, which applied the more conservative RIFLE criteria in a cohort without diabetes [19].

### Incidence of AKI compared to controls

A significantly lower incidence of AKI was reported in three studies: Landoni 26.9% vs. 31.7%; RR 0.85 (95%CI 0.77–0.94;*p* = 0.002), Holm 4.9% vs. 10.0%; RR 0.49 (95%CI 0.29–0.83; *p* = 0.008) and Kazawa 30.3% vs. 56.2%; RR 0.63 (95% CI, 0.40–0.99; *p* = 0.04) or adjusted HR 0.44 (0.20–0.95: *p* = 0.04). Data for the remaining trials are summarized in Table 5.

## Concluding remarks

This overview identified significant heterogeneity among studies, as detailed in Tables 3, 4 and 5. Heterogeneity per se does not necessarily undermine the validity of the meta-analyses but has to be considered while interpreting the data.

RIFLE, AKIN and KDIGO (Table 1) are composite endpoints comprising criteria that do not carry equal clinical weight, which makes them problematic for scientific study purposes [80]. Wiersema et al. reported that KDIGO offers multiple options for defining AKI in ICU patients, resulting in substantial variability in the incidence of AKI [81]. The study by Kazawa et al. was the only one to apply the full KDIGO criteria, and the significant outcome was primarily driven by urine output data, which may facilitate early identification of AKI but has uncertain prognostic value [14, 69].

The most evident caveats relate to differences in the proposed mechanisms, dosing regimens, and amino acid selection. Across the trials, three distinct mechanisms were claimed to explain how amino acids could prevent AKI in cardiac surgery. The study by Weiss et al. was based on the putative general beneficial properties of glutamine in critically ill patients, including protection against inflammation and ischemia-reperfusion injury [61]. The other trials were based on more specific mechanistic hypotheses. Landoni (PROTECTION), Pu and Kazawa targeted recruitment of RFR through amino acid loading, whereas Holm (GLUTAMICS I-II) proposed that glutamate prevents AKI indirectly by enhancing post-ischemic recovery of the heart [8, 10, 14, 19].

The amino acid dose in PROTECTION (Landoni et al.) was at least 15-fold higher and the infusion lasted 1–3 days, compared with 2.5–3 h in GLUTAMICS I-II (Holm et al.) [10, 19]. The amino acid mixture used in PROTECTION did not contain glutamate. These two trials accounted for more than 90% of the patients in these five trials (Table 3). However, only the PROTECTION trial qualifies as an adequate RCT.

Preserved kidney function is necessary for recruitment of RFR, and the observed benefit was confined to NYHA class I-II patients. Although this may partly reflect the smaller number of NYHA class III–IV patients, it remains unclear whether amino acid loading improves long-term outcomes or benefits patients with advanced disease. The editorial by Ostermann et al. concluded that positive effects from large RCTs like PROTECTION should drive clinical change but questioned to what extent the results reflected a functional effect or proper protection against tubular kidney injury. Future trials were recommended to include biomarkers of tubular kidney injury and long-term follow-up of kidney function [35]. Subsequent post hoc analyses from the PROTECTION trials suggest that amino acid loading is cost-effective and reduces the incidence of AKI in patients with prolonged CPB time and in those requiring temporary mechanical circulatory support [37, 82, 83].

AKI was not a prespecified primary endpoint in either of the two GLUTAMICS trials, and the results from GLUTAMICS I–II were derived from a post hoc pooled analysis. Glutamate infusion was associated with a halved incidence of AKI, defined as RIFLE stage I or higher (Table 5) [19, 53, 54]. The reduction in more severe AKI (RIFLE stage II or higher) appeared greater but should be interpreted cautiously due to the limited number of events. The benefit was most evident in high-risk patients, supporting further evaluation of intravenous glutamate infusion in high-risk patients without diabetes undergoing CABG [53, 54].

Some limitations warrant consideration. As a non-systematic narrative review, it is inherently more prone to bias than a systematic review. The focus is on trials of amino acid infusion aimed at preventing AKI in cardiac surgery. While key background information on different strategies is provided, preclinical and observational data are not comprehensively addressed.

Approximately two million cardiac surgical procedures are performed worldwide each year, and AKI remains one of the most common serious complications [29]. Amino acid infusions have emerged as a promising strategy for AKI prevention; however, the number of randomized clinical trials is limited [17]. Nevertheless, based on the PROTECTION trial and available meta-analyses, intravenous amino acids should be considered for renoprotection, consistent with the recently issued Class IIa recommendation in the EACTS/EACTAIC/EBCP 2024 Guidelines on cardiopulmonary bypass in adult cardiac surgery [84]. Additional clinical trials are warranted to provide more robust evidence on long-term outcomes and protection against tubular kidney injury. Future research should also address key knowledge gaps, including oral versus intravenous administration, timing, optimal amino acid selection and dosing, and the patient populations most likely to benefit.

## Data Availability

Not applicable.

## Abbreviations

AA: Amino Acids
AKI: Acute Kidney Injury
AKIN: Acute Kidney Injury Network
BW: Body Weight
CABG: Coronary Artery Bypass Grafting
CCS class: Canadian Cardiovascular Society grading of angina pectoris
CI: Confidence Interval
CPB: Cardiopulmonary Bypass
EAAT1: Excitatory Amino Acid Transporter 1
EuroSCORE II: European System for Cardiac Operative Risk Evaluation II
GFR: Glomerular Filtration Rate
GLUTAMICS: GLUTAmate for Metabolic Intervention in Coronary Surgery
ICU: Intensive Care Unit
KDIGO: Kidney Disease Improving Global Outcomes
KIM^1^: Kidney Injury Molecule-1
KRT: Kidney Replacement Therapy
NO: Nitric Oxide
NGAL: Neutrophil Gelatinase-Associated Lipocalin
NT-proBNP: N-Terminal pro-Brain Natriuretic Peptide
NYHA: New York Heart Association
RCT: Randomized Controlled Trial
RFR: Renal Functional Reserve
RIFLE: Risk, Injury, Failure, Loss, and End stage criteria
s-Cr: serum Creatinine
[TIMP2]*[IGFBP7]: [Tissue Inhibitor Metallo Proteinase 2]*[Insulin-like Growth Factor-Binding Protein 7]
